# Nrf2 attenuates ferroptosis-mediated IIR-ALI by modulating TERT and SLC7A11

**DOI:** 10.1038/s41419-021-04307-1

**Published:** 2021-10-29

**Authors:** Hui Dong, Yangyang Xia, Shanliang Jin, Chaofan Xue, Yanjun Wang, Rong Hu, Hong Jiang

**Affiliations:** 1 Shanghai Ninth People’s Hospital, Shanghai JiaoTong University School of Medicine, Center for Specialty Strategy Research of Shanghai Jiao Tong University China Hospital Development Institute, Shanghai, China; 2 Shanghai Synchrotron Radiation Facility, Shanghai Advanced Research Institute, Chinese Academy of Sciences, Shanghai, China

**Keywords:** Respiratory tract diseases, Acute inflammation

## Abstract

Acute lung injury (ALI) carries a mortality rate of ~50% and is a hot topic in the world of critical illness research. Nuclear factor erythroid 2-related factor 2 (Nrf2) is a critical modulator of intracellular oxidative homeostasis and serves as an antioxidant. The Nrf2-related anti-oxidative stress is strongly associated with ferroptosis suppression. Meanwhile, telomerase reverse transcriptase (TERT), the catalytic portion of the telomerase protein, is reported to travel to the mitochondria to alleviate ROS. In our study, we found that TERT was significantly reduced in lung tissue of Nrf2^−/−^ mice in the model of intestinal ischemia/reperfusion-induced acute lung injury (IIR-ALI). In addition, MDA levels showed marked increase, whereas GSH and GPX4 levels fell drastically in ALI models. Moreover, typical-related structural changes were observed in the type II alveolar epithelial cells in the IIR model. We further employed the scanning transmission X-ray microscopy (STXM) to examine Fe levels and distribution within cells. Based on our observations, massive aggregates of Fe were found in the MLE-12 cells upon OGD/R (oxygen and glucose deprivation/reperfusion) induction. Additionally, Nrf2 silencing dramatically reduced TERT and SLC7A11 levels, and further exacerbated cellular injuries. In contrast, TERT-overexpressing cells exhibited marked elevation in SLC7A11 levels and thereby inhibited ferroptosis. Collectively, these data suggest that Nrf2 can negatively regulate ferroptosis via modulation of TERT and SLC7A11 levels. The conclusion from this study brings insight into new candidates that can be targeted in future IIR-ALI therapy.

## Introduction

Ischemia reperfusion (IR) injury is a potential surgical complication that can inadvertently release cytotoxic agents from ischemic tissues and inflammatory mediators and produce secondary vascular disease [1]. This may ultimately result in sepsis, systemic inflammatory response syndrome, and multiple organ dysfunction syndrome [2]. Intestinal ischemia reperfusion (IIR) can also lead to acute lung injury (ALI) and can ultimately cause acute respiratory distress syndrome. It carries a high mortality rate and is a major challenge to the world of critical illness research [3–6].

Ferroptosis is a critical form of cell death that involves destruction of the intracellular anti-oxidative process and accumulation of massive amounts of reactive oxygen species (ROS) in the mitochondria [7–10]. Furthermore, elevated ferroptosis was shown to exasperate visceral dysfunction in IR-induced renal and liver injury models [11, 12]. Unfortunately, few studies have investigated the role of ferroptosis in the distribution and content of iron (Fe) in the alveolar epithelial cells during IIR-induced ALI (IIR-ALI).

Nuclear factor E2 related factor 2 (Nrf2) is a critical modulator of oxidative homeostasis and is produced in response to elevated oxidative stress. Nrf2 interacts with the antioxidant response element (ARE) in the nucleus and exerts cellular protection by targeting gene transcription and protein translation of antioxidant and anti-inflammatory proteins [13–15]. It, therefore, regulates critical cellular defense mechanisms and offers protection against cerebral [16], liver [17], cardiac [18], and IIR damage [19, 20]. In a recent study, Nrf2 silencing was shown to reverse ferroptosis resistance normally seen with anti-tumor drug therapy [21–23]. But, its underlying mechanisms of ferroptotic regulation are not fully understood.

Telomerase reverse transcriptase (TERT) is a rate-limiting enzyme involved in telomere maintenance [24]. Overexpression of the human TERT (hTERT) was shown to reduce oxidative stress-induced intracellular ROS levels via elevation of the reduced/oxidized glutathione ratio, thereby improving mitochondrial function and cell survival [25]. However, whether TERT exerts an indispensable effect on ferroptosis remains unknown.

Here, we examined cellular ferroptosis by exploring Fe distribution and content in vitro IIR-ALI models, via a new detection technique called scanning transmission X-ray microscopy (STXM). We speculated that Nrf2 silencing during ischemia induces ferroptosis via TERT and SLC7A11 modulation. In addition, we evaluated whether TERT protected against ferroptosis normally seen with IIR-ALI, via regulation of SLC7A11. Our research will uncover potential therapeutic targets in the management of IIR-ALI.

## Results

### II/R-induced ALI promotes ferroptosis

**Fig. 1:**
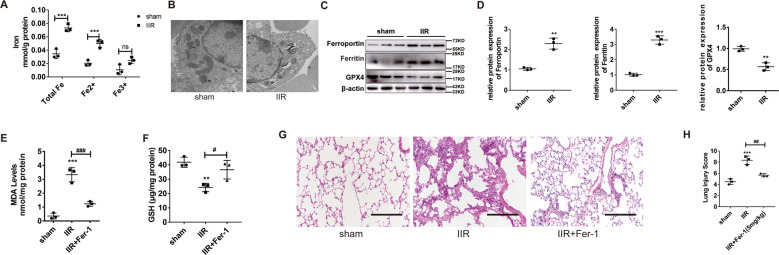
II/R-induced ALI promotes ferroptosis. **A** Endogenous Fe levels in the lungs of IIR-ALI mice, as evidenced by Fe assay. **B** Representative transmission electron micrographs of lung ultrastructure. Scale bars: 500 nm. **C**, **D** Evaluation and quantification of the GPX4, ferritin, and ferroportin protein expressions, under all conditions. **E** Evaluation of the lipid peroxide MDA levels under all conditions. **F** GSH levels under all conditions. **G** HE staining of the murine lung tissues after IIR and IIR + Fer-1. Scale bars: 200 μm. **H** The lung pathological damage score illustrating addition and decrease after Fer-1 administration, respectively. Data are expressed as mean ± SEM. *P < 0.05; **P < 0.01; ***P < 0.001. Error bars denote standard error from three individual experiments.

To explore the role of ferroptosis in the IIR-ALI model, we first detected the endogenous ferric and ferrous levels in the lung tissues of the IIR-ALI model. Total Fe, Fe^2+^, and Fe^3+^ levels in the IIR mice were markedly higher, relative to the sham mice (Fig. 1A). Additionally, using TEM, we showed that, relative to the sham mice, the type II alveolar epithelial cells of IIR mice exhibited characteristics typical of ferroptosis, namely, reduced mitochondrial size and smaller cristae (Fig. 1B). In addition, the GPX4 (critical enzyme regulating lipid peroxidation) levels were markedly diminished, whereas the ferritin and ferroportin (critical proteins of ferroptosis) levels were strongly elevated in the IIR model versus sham mice (Fig. 1C, D). Using hematoxylin and eosin (HE) staining (Fig. 1G), we further demonstrated that IIR produced significant damage to the lung tissue (*P* < 0.05). Moreover, the lipid peroxide MDA levels rose (Fig. 1E), whereas the reduced glutathione (GSH) levels fell (Fig. 1F), which again is highly characteristic of the ferroptosis process. To further examine ferroptosis activation in the IIR model, we introduced Fer-1, a specific inhibitor, into the murine tail vein and harvested lung tissue at the end of the experiment. We revealed via HE staining (Fig. 1G) that Fer-1 dramatically decreased the severity of damage in the IIR mice lung tissue, compared to untreated IIR mice. Simultaneously, the MDA levels (Fig. 1E) diminished and GSH increased with Fer-1 administration, relative to the untreated IIR mice (Fig. 1F). Taken together, these data indicate that IIR-ALI mice exhibit accelerated ferroptosis, which, in turn, aggravate severity of the disease.

### Nrf2 knockout accelerates ferroptosis and aggravates IIR-ALI by regulating TERT and SLC7A11

**Fig. 2:**
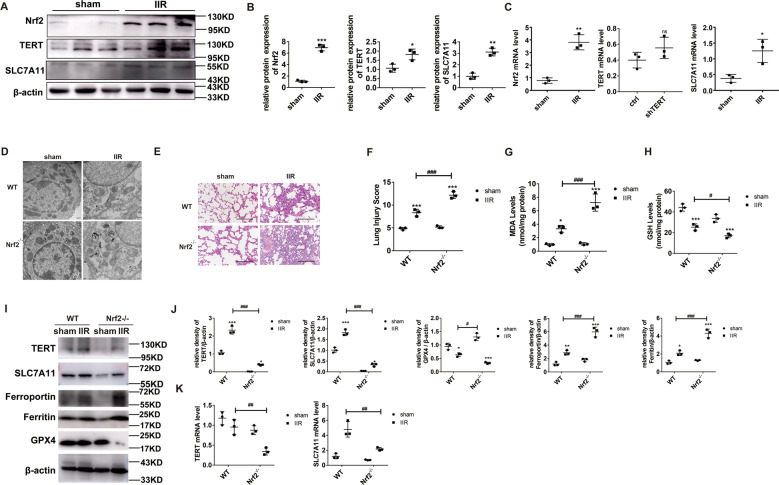
The Nrf2/TERT/SLC7A11 axis is activated during II/R and the ferroptotic process is dysregulated in the Nrf2^−/−^ mice after II/R. **A** Evaluation of Nrf2 and its potential downstream targets in the murine lung tissues, under different conditions, as evidenced by western blotting. **B** Quantification of the examined proteins. **C** mRNA levels of the examined factors. **D** Representative transmission electron micrographs of the lung tissue ultrastructures. Scale bar: 500 nm. **E** HE staining of lung sections, observed under a light microscope. Scale bar: 200 μm. **F** Pathological scores, as determined by an experienced pathologist. **G** MDA levels under different conditions. **H** GSH levels under different conditions. **I** Evaluation of essential proteins involved in ferroptosis regulation within lung tissues, under different conditions. **J** Quantification of the examined proteins. **K** mRNA levels of the examined factors. Error bars denote standard error from three individual experiments. Data are expressed as mean ± SEM. *P < 0.05; **P < 0.01; ***P < 0.001. # relative to the IIR-WT mice.

To delineate the Nrf2-mediated regulation of ferroptosis and IIR-ALI, we examined the Nrf2 gene knockout (Nrf2^−/−^) mice. Nrf2 is an essential modulator of anti-oxidative stress. Hence, Nrf2 levels were markedly elevated in the IIR model versus sham mice (Fig. 2A). Using TEM, we showed that the mitochondria was small in size, the cristae was either reduced or absent, and the outer membrane was torn in the Nrf2^−/−^ IIR mice versus WT mice (Fig. 2D). This indicates accelerated ferroptosis in the absence of Nrf2. Likewise, the IIR-ALI pathology was more severe in the Nrf2^−/−^ IIR versus WT mice (Figs. 2E, F), suggesting that Nrf2 offers protection against ALI, which is in line with other studies [17]. Moreover, we demonstrated strong GSH suppression in the IIR model, which was more prominent in the Nrf2^−/−^ mice (Fig. 2H) and we observed an elevation in MDA levels in the IIR model, which was more prominent in the Nrf2^−/−^ mice (Fig. 2G). Additionally, GPX4 levels, which were low in the IIR model, were even lower in the Nrf2^−/−^ mice, whereas critical Fe metabolic proteins ferritin and ferroportin were high in the IIR model and were present in even higher concentrations in the Nrf2^−/−^ mice (Fig. 2I, J). Collectively, these results suggest that the Nrf2^−/−^ mice lost their ability to modulate ferroptosis following II/R.

IIR-ALI induction dramatically increased the levels of total Nrf2, TERT, and SLC7A11 protein and mRNA (Fig. 2A–C). To elucidate the Nrf2 regulation of these proteins, we examined their expression in the Nrf2^−/−^ mice. Using western blotting, we demonstrated that TERT and SLC7A11 fell dramatically in the Nrf2^−/−^ versus WT mice (Fig. 2I–K). Based on these data, it is possible that Nrf2 negatively regulates cellular ferroptosis and IIR-ALI via modulation of TERT and SLC7A11 levels.

### Ferroptosis boosts OGD/R-driven lung epithelial cell damage

**Fig. 3:**
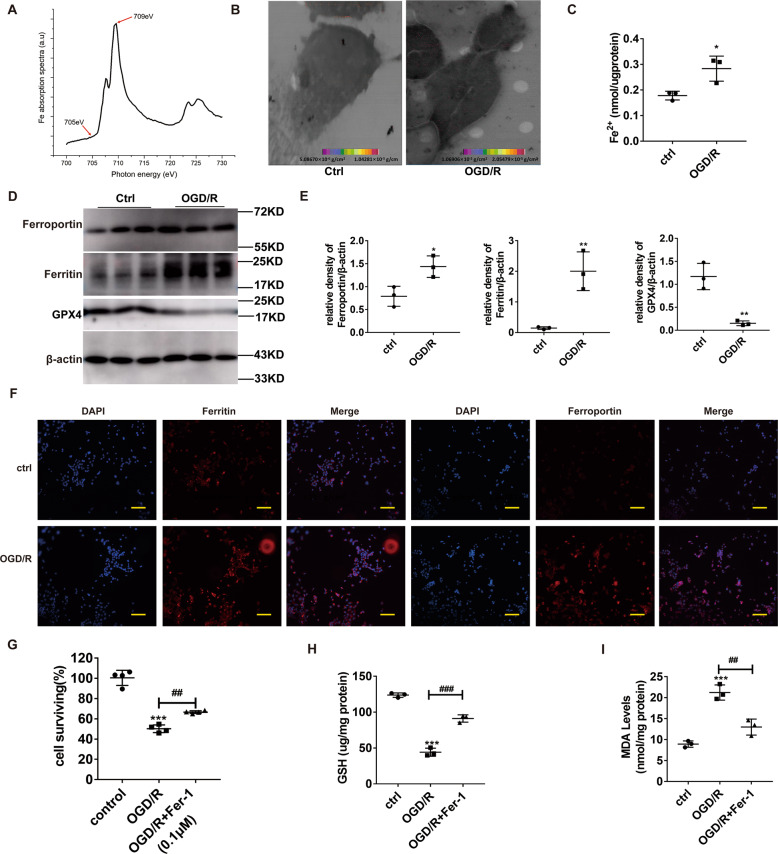
OGD/R accelerates ferroptosis within pulmonary epithelial cells and enhances Nrf2, TERT, and SLC7A11 levels during ferroptosis. **A** Fe L3-edge NEXAFS (near edge X-ray absorption fine structure) spectrum was acquired from two absorption cellular images scanned at two energies, *E*1 (709 eV) and *E*2 (705 eV) above and below the absorption edge of Fe, respectively. **B** Evaluation of Fe distribution and content using scanning transmission X-ray microscopy. **C** Endogenous Fe levels within the pulmonary epithelial cells of the OGD/R model, as detected by Fe assay. **D** Evaluation of the levels of ferroptosis-related proteins under different conditions. **E** Quantification of the examined proteins. **F** Evaluation of ferritin and ferroportin levels (red) in lung tissues, using immunohistochemistry. Nuclear staining done with DAPI (blue) after OGD/R. Scale bar: 100 μm. **G** Cell survival after OGD/R induction and the opposite effect with Fer-1 (0.1 μM). **H** GSH levels under different conditions. **I** MDA levels under different conditions. Error bars denote standard error from three individual experiments. Data are expressed as mean ± SEM. *P < 0.05; **P < 0.01; ***P < 0.001; *relative to control; ^#^relative to OGD/R.

To validate our former data, we employed the MLE12 cell line to analyze the distribution and content of cellular Fe, using STXM. We scanned two absorption cell images at two separate energies, namely, E1 (709 eV) and E2 (705 eV), above and below the Fe absorption edge, respectively (Fig. 3A). Based on the different cellular absorption rates to incident light at these energies, we obtained the cellular Fe distribution and surface density information by comparing the OD of the corresponding pixel points in both images. We, thus, showed that the Fe surface density in control cells was 5.09 × 10^−6^ to 1.04 × 10^−5^ g/cm^2^, while that in the OGD/R group was 1.07 × 10^−5^ to 2.05 × 10^−5^ g/cm^2^. Compared to the control cells, Fe aggregated in large amounts within the MLE-12 cells after OGD/R induction, and its content was also significantly increased (Fig. 3B). In addition, the endogenous Fe level in MLE-12 cells was tested in both the control and OGD/R group. We observed remarkably higher Fe in the OGD/R group, as opposed to the control (Fig. 3C). Next, we tested the expression of ferritin, ferroportin, and GPX4 proteins. OGD/R markedly downregulated GPX4 (Fig. 3D) and upregulated ferritin and ferroportin levels (Fig. 3D, F). Moreover, OGD/R dramatically reduced the quantity of adherent cells (all *P* < 0.001) (Fig. 3G) and levels of GSH (Fig. 3H), while increasing MDA content (Fig. 3I). Moreover, these oxidative stress-related markers were restored to normal levels via co-incubation of MLE12 cells with Fer-1 (0.1 μM) (Fig. 3G–I). In all, these data suggest that OGD/R promotes ferroptosis-mediated cell death in pulmonary epithelial cells.

### Nrf2 deficiency downregulates TERT and SLC7A11, and produces more extensive ferroptosis after OGD/R induction

**Fig. 4:**
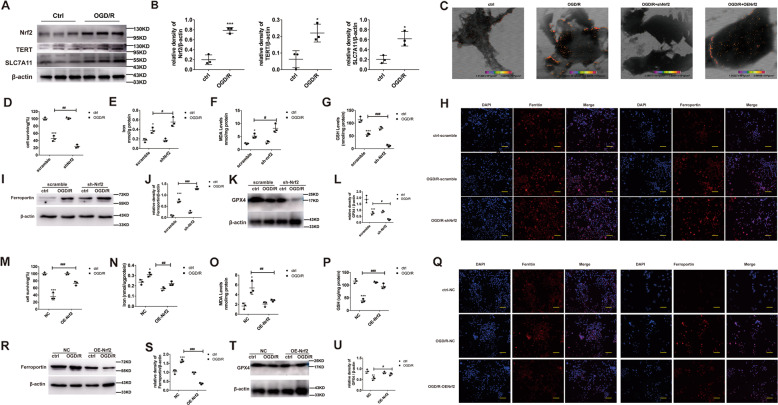
Nrf2 stimulation prevents ferroptosis and the absence of Nrf2 produces more serious ferroptosis phenotype in OGD/R. **A** Evaluation of Nrf2 and its potential downstream proteins via western blotting, under different conditions. **B** Quantification of the examined proteins. **C** Fe distribution and content detected via scanning transmission X-ray microscopy. **D**, **M** Evaluation of cell viability after OGD/R induction, as evidenced by CCK8 assay. Cells were assigned to three categories: (1) control, (2) OGD/R, and (3) OGD/R + Fer-1 (0.1 μM). **E**, **N** Endogenous Fe levels within pulmonary epithelial cells in the OGD/R model, as detected by Fe assay. **F**, **O** MDA levels after OGD/R induction, as assessed by MDA assay. **G**, **P** GSH levels after OGD/R induction, as assessed by GSH assay. **H**, **Q** Evaluation of ferritin and ferroportin (red), using immunohistochemistry. Nuclear staining done with DAPI (blue) after OGD/R induction. Scale bar: 100 μm. **I–L, R**–**U** Evaluation and quantification of ferroportin and GPX4 protein levels. Error bars denote standard error from three individual experiments. Data are expressed as mean ± SEM. *P < 0.05, **P < 0.01, ***P < 0.001 represent intergroup significant differences. *Relative to the sham control mice. ^#^Relative to the OGD/R control.

**Fig. 5:**
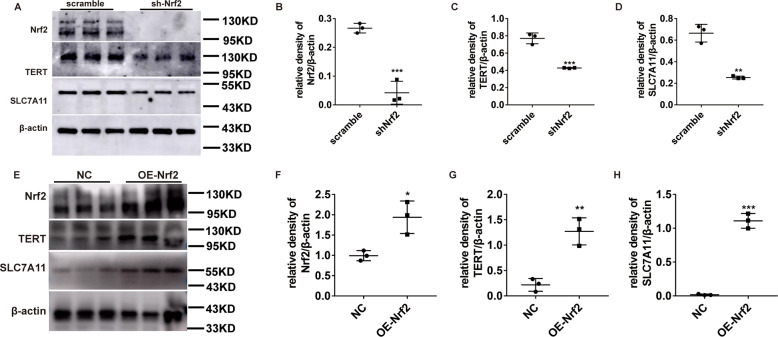
Nrf2 positively regulates TERT and SLC7A11 levels during OGD/R-driven ferroptosis in pulmonary epithelial cells. **A**, **E** Evaluation of Nrf2 and its potential downstream signaling proteins via western blotting, under different conditions. **B–D**, **F–H** Quantification of the examined proteins. Error bars denote standard error from three individual experiments. Data are expressed as mean ± SEM. *P < 0.05, **P < 0.01, ***P < 0.001 represent intergroup significant differences.

Consistent with our in vivo studies, the Nrf2/TERT/SLC7A11 expression was dramatically increased after OGD/R induction in the MLE-12 cells (Fig. 4A, B). To further explore the signaling pathways mediating Nrf2 regulation of ferroptosis and ALI, these cells were infected with the Nrf2-shRNA lentivirus. We demonstrated that Fe formed aggregates in MLE-12 cells after OGD/R induction, as opposed to non-induced MLE-12 cells. Moreover, its content significantly increased in the shNrf2 group, and could be reversed by overexpressing Nrf2. (Fig. 4C). Next, we examined the levels of oxidative stress markers, namely, CCK8, GSH, MDA, and Fe^2+^. The cell viability and GSH percentage reduced further, whereas the percentage of MDA and Fe^2+^ levels augmented further (Fig. 4D–G). In addition, with our evaluation of the ferritin, ferroportin, and GPX4 proteins, we demonstrated that ferroptosis and oxidative stress were significantly more evident in the shNrf2-treated cells versus scramble-treated cells (Fig. 4H–L). Taken together, the shNrf2-treated cells exhibited accelerated ferroptosis, relative to the scramble-treated cells. Conversely, Nrf2 overexpression with a lentivirus significantly increased cell viability and GSH content, and the MDA content and Fe^2+^ levels significantly decreased (Fig. 4M–P). Moreover, evaluation of the ferritin, ferroportin, and GPX4 proteins (Fig. 4Q–U) showed that ferroptosis and oxidative stress were dramatically reduced in the OENrf2-treated cells, compared to the NC-treated cells. Interestingly, we observed that TERT and SLC7A11 expression was markedly reduced in the shNrf2-treated cells and increased in the OENrf2-treated cells, suggesting that Nrf2 can indeed suppresses ferroptosis and abrogate ALI, and this process involves regulation of the TERT and SLC7A11 proteins (Fig. 5A–H).

### Overexpression of TERT (OETERT) alleviates ferroptosis via modulation of SLC7A11

**Fig. 6:**
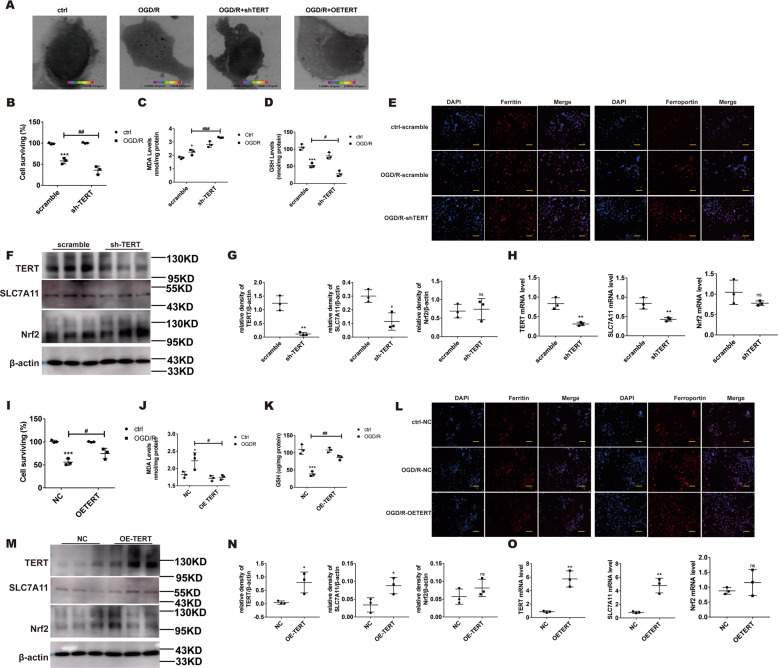
Low TERT levels enhance ferroptosis, whereas TERT overexpression (OETERT) alleviates ferroptosis via upregulating SLC7A11. **A** Fe distribution and content. **B**, **I** Evaluation of cell viability after OGD/R, as evidenced by CCK8 assay. **C**, **J** MDA levels after OGD/R, as assessed by MDA assay. **D**, **K** GSH levels after OGD/R, as assessed by GSH assay. **E**, **L** Evaluation of ferritin and ferroportin (red), using immunohistochemistry. Nuclear staining done with DAPI (blue) after OGD/R. Scale bar: 100 μm. **F**, **M** Evaluation of Nrf2 and its potential downstream proteins via western blotting under different treatment conditions. **G**, **N** Protein quantification. **H**, **O** mRNA levels of the examined factors. Error bars denote standard error from three individual experiments. Data are expressed as mean ± SEM. *P < 0.05, **P < 0.01, ***P < 0.001 denotes intergroup significant difference. *Relative to the sham control mice. ^#^Relative to the OGD/R controls.

To delineate the TERT-mediated regulation of IIR-driven ALI, MLE-12 cells were infected with either TERT-shRNA lentivirus or TERT-overexpressing lentivirus. We demonstrated a large presence of Fe aggregates in the OGD/R group, relative to controls. More importantly, its content was significantly increased in the shTERT group, and was reversed by TERT overexpression (Fig. 6A). We also assessed the relative levels of CCK8, GSH, and MDA. Based on our data, the cell viability and GSH levels experienced further decrease and MDA levels saw further increase in the OGD/R shTERT-incorporated cells versus OGD/R scramble-incorporated cells (Fig. 6B–D). Next, using immunohistochemical staining lung tissues (Fig. 6E), we demonstrated that ferroptosis was significantly greater in the shTERT OGD/R-incorporated cells, as opposed to the scramble OGD/R-incorporated controls. Moreover, TERT overexpression significantly reversed all the above changes (Fig. 6I–L), indicating that both ferroptosis and oxidative stress were drastically alleviated after OETERT OGD/R incorporation versus NC OGD/R incorporation. We further explored the relationship between TERT and SLC7A11 proteins via western blotting and quantitative real-time PCR (QPCR). Based on our results, SLC7A11 were significantly reduced in the shTERT-infected cells and increased in the OETERT-infected cells. However, there was no discernible change in Nrf2 expression following TERT expression (Fig. 6F–H, M–O), thereby indicating that the anti-ferroptotic role of TERT may be exerted via modulation of SLC7A11 levels after OGD/R induction.

### TERT inhibited ferroptosis-dependent OGD/R injury by facilitated SLC7A11 expression in vitro

**Fig. 7:**
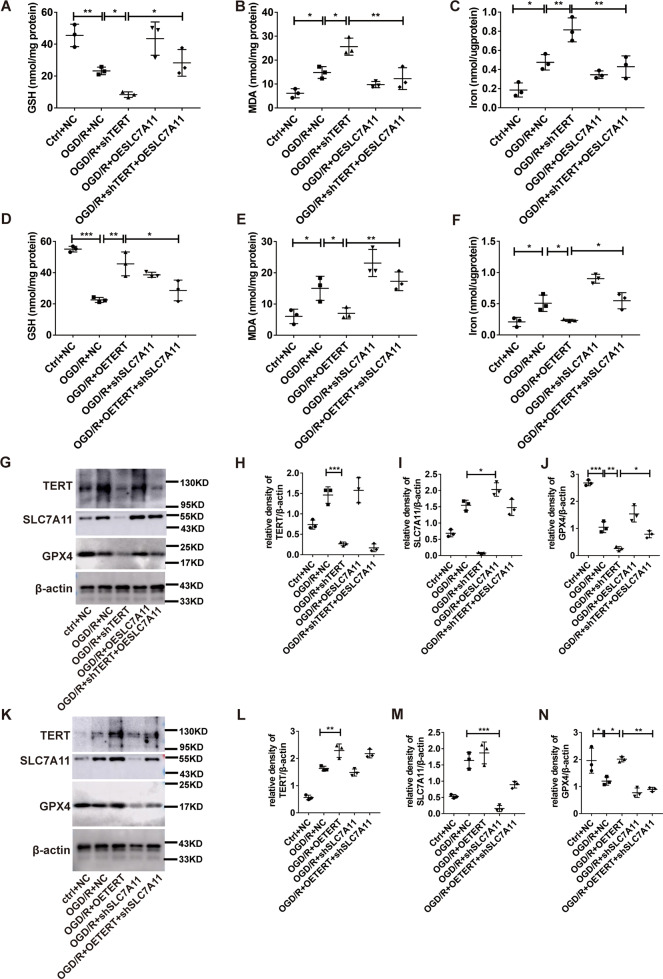
TERT inhibited ferroptosis-dependent OGD/R injury by facilitated SLC7A11 expression in vitro. **A, D** GSH levels after OGD/R, as assessed by GSH assay. **B**, **E** MDA levels after OGD/R, as assessed by MDA assay. **C**, **F** Endogenous Fe levels within pulmonary epithelial cells in the OGD/R model, as detected by Fe assay. **G**, **K** Evaluation of TERT, SLC7A11, and GPX4 protein expression via western blotting under different treatment conditions. **H–J**, **L–N** Protein quantification. Data are expressed as mean ± SEM. *P < 0.05, **P < 0.01, ***P < 0.001 denotes intergroup significant difference.

In order to further verify whether TERT exerts its anti-ferroptosis effect through SLC7A11, we down regulate TERT and overexpress SLC7A11 in MLE cells simultaneously. We found that overexpression of SLC7A11 can rescue the pro-ferroptotic effect of inhibiting TERT by facilitating the expression of GSH and GPX4, while reducing the levels of MDA and iron content (Fig. 7A–C, G, J). On the contrary, when OETERT and shSLC7A11 lentiviruses were transfected into MLE at the same time, downregulating SLC7A11 could eliminate the inhibitory anti-ferroptotic effect of overexpression TERT by inhibiting the expression of GSH and GPX4, while increasing the levels of MDA and iron content (Fig. 7D–F, K, N).

Taken together, these data suggest that Nrf2 offers protection against IIR-ALI via ferroptosis regulation, which is mediated by the modulation of TERT and SLC7A11 levels.

## Discussion

Ferroptosis is a newly discovered type of programmed cell death [26] that involves lipid peroxidation as well as Fe and ROS accumulation. Emerging reports indicate a strong correlation between ROS and IIR pathology. We, therefore, speculated that IIR or OGD/R may be essential to the promotion of ferroptosis [27]. To test this, we examined the relative levels of oxidative stress markers like GSH, MDA, and Fe^2+^. We also detected the expression of iron metabolism-related proteins, such as ferritin and ferroportin. Ferritin is found in most tissues that store and keep iron in a soluble and nontoxic form [28]. It also represents an indirect marker of the total amount of intracellular iron, which increases with iron overload. Ferroportin is the only known mammalian iron exporter [29] and a transmembrane protein that transfers iron from cells, such as enterocytes, liver, spleen to plasma [30]. Studies have shown that the intraperitoneal iron overload in mice results in an increase in ferroportin mRNA expression and protein levels [31], and an increase in ferroportin expression is an indicator of iron accumulation in hepatic Kupffer cells [32]. Based on our results, the GSH percentage reduced dramatically and the MDA percentage, along with Fe^2+^ levels, showed a marked elevation. Moreover, evaluation of the ferroptosis-related proteins ferritin, ferroportin, and GPX4 suggested that both ferroptosis and oxidative stress were markedly elevated in the IIR and OGD/R groups versus controls. Simultaneously, we observed ferroptosis-related mitochondrial structural changes in the type II alveolar epithelial cells of IIR mice. Hence, the epithelium experienced ferroptosis during IIR.

Previous studies have not reached a general consensus on an effective way of measuring Fe distribution and content within MLE-12 cells. In this study, we utilized STXM to perform this analysis. STXM evaluation was performed at both above and below the L3 absorption edge of Fe, namely, 709 and 705 eV, respectively. Furthermore, Fe distribution and surface density information was obtained by comparing the OD of the corresponding pixel points in the two images, based on the different cellular absorption rates in response to incident light at different energies. Based on our results, compared to the controls, the OGD/R group exhibited vastly increased Fe aggregates and contents.

To elucidate the ferroptosis-mediated regulation of IIR-ALI, Fer-1 was administered via the murine tail vein. Being a strong ferroptosis suppressor, Fer-1 alleviated lung damage via suppressing lipid peroxidation and enhancing epithelial cell viability. Hence, we propose that IIR-ALI and OGD/R conditions accelerate ferroptosis, which, in turn, aggravates both lung and cellular destruction. Therefore, in contrast, ferroptosis suppression prevents this lethal cascade.

Nrf2 is a critical responsive transcription factor of oxidative stress that stimulates downstream pathways that regulate ferroptosis via modulation of glutathione, Fe, and lipid metabolism, as well as mitochondrial function [33]. Moreover, Nrf2 stimulation was reported to accelerate cancer cell proliferation and abrogate response to ferroptosis activators [21, 34]. However, the exact role of Nrf2 on the ferroptosis associated with IIR-ALI is not yet known. In a prior study, we demonstrated marked Nrf2 upregulation in a IIR-ALI mouse model [35]. We speculated that the Nrf2 role as an anti-oxidative modulator could potentially prevent ferroptosis, thereby alleviating IIR-ALI [24]. Here, we revealed that Nrf2 overexpression lead to a marked protection against ferroptosis damage in the IIR-ALI models. Moreover, the Nrf2^−/−^ mice under IIR-ALI conditions exhibited markedly more elevation in oxidative stress markers, namely, MDA and Fe^2+^, with a substantial further decrease in GSH levels, compared to the WT IIR mice. Additionally, examination of the ferroptosis-related proteins ferritin, ferroportin, and GPX4 suggested that ferroptosis and oxidative stress were significantly enhanced in the Nrf2^−/−^ mice, as opposed to the WT mice. At the same time, we found that Nrf2 negatively regulates ferritin and ferroportin, which may be due to the further progress or alleviation of ferroptosis after interference or overexpression of Nrf2, thus make these two proteins undergo compensatory changes to maintain the stability of iron pool. Taken together, these data suggest that Nrf2 deficiency can further aggravate IIR-driven lung injury via ferroptosis stimulation. Moreover, interestingly, TERT levels showed a marked decrease in the Nrf2^−/−^ IIR mice, compared to WT IIR mice, suggesting a possible TERT involvement in the regulation of ferroptosis and IIR-ALI.

*Tert* is a key gene regulating telomerase activity, which plays a key role in IR disease [24]. More and more evidences suggest that hTERT regulates cellular redox metabolic processes. But, no direct link has yet been discovered between TERT and ferroptosis. In this study, TERT expression was significantly reduced in the lung tissues of Nrf2^−/−^ mice with IIR, suggesting that TERT may be under Nrf2 regulation and may reduce cell injury via modulation of ROS levels. Additionally, TERT overexpression was shown to prevent MLE12 cellular injury, reduce Fe accumulation, and levels of ferroptosis-related proteins, while promoting levels of SLC7A11. In contrast, TERT inhibition was shown to reverse all the above-mentioned changes, thus demonstrating that TERT protected against OGD/R-driven ferroptosis.

SLC7A11 is a specific light chain subunit of the cystine/glutamate antiporter, which serves as an inverse ferroptosis modulator that sustains a homeostatic redox state [15]. Sun et al. [21] reported that Fe modulates SLC7A11 expression via the ROS–Nrf2 axis. Here, both TERT and SLC7A11 levels were elevated under IIR and OGD/R conditions, suggesting that they are intricately involved in the IIR-ALI-mediated regulation of ferroptosis. In addition, using *Nrf2* silencing, we demonstrated that the TERT and SLC7A11 proteins were markedly downregulated, thus enabling lipid peroxide accumulation. Collectively, these data demonstrate that Nrf2 serves its anti-ferroptosis function via modulation of TERT and SLC7A11 expression.

To further examine the regulation between TERT and SLC7A11, we employed lentiviral infection to either overexpress or suppress TERT levels in MLE-12 cells. We demonstrated that overexpressing TERT increased the production of SLC7A11, whereas TERT deficiency dramatically decreased SLC7A11 generation. Additionally, we transfected shTERT and OESLC7A11 lentiviruses into MLE cells at the same time, and found that overexpression of SLC7A11 can rescue the pro-ferroptotic effect of inhibiting TERT by facilitating the expression of GSH and GPX4, while reducing the levels of MDA and iron content. On the other hand, when OETERT and shSLC7A11 lentiviruses were transfected into MLE simultaneously, shSLC7A11 could eliminate the anti-ferroptotic effect of OETERT. This suggests a modulatory role of TERT on ferroptosis via SLC7A11.

In conclusion, we used the Nrf2^−/−^ mice and the MLE-12 cell line to establish the IIR and OGD/R model. Moreover, we utilized the Shanghai light source device of the synchrotron radiation soft X-ray spectroscopy microbeamline, with the synchrotron radiation soft X-ray spectroscopy microscopic imaging technique, to observe Fe distribution and content in MLE-12 cells and examine the Nrf2/TERT/SLC7A11 axis of ferroptosis regulation and IIR-ALI alleviation. Based on our results, we propose that Nrf2 offers protection against IIR-ALI via modulating TERT and SLC7A11 levels, and ultimately ferroptosis. Our work has great therapeutic potential in the treatment of II/R-driven ALI.

## Materials and methods

### Animals

We received 60 C57BL/6J mice and 48 Nrf2 knockout (Nrf2^−/−^) mice of the same genetic background from the RIKEN Bio-Resource Centre via the National BioResource Project, MEXT, Japan. All animals were 8 weeks old and were maintained at 21 ± 2 °C with a humidity of 60 ± 5% and a 12 h light/12 h dark cycle. They received standard mouse chow and water ad libitum. Our animal protocols followed the strict NIH guidelines and received ethical approval from the Shanghai Ninth People’s Hospital for Animal Research.

### IIR mouse model

Animals were placed on a fast, with free access to water, for 24 h prior to experimentation. Intestinal ischemia was simulated by clamping the superior mesenteric artery following the intraperitoneal administration of 50 mg/kg sodium pentobarbital. Forty-five minutes later, the intestine was allowed to re-perfuse for 3 h. Sham control mice received the same treatment, except for the vascular occlusion. In some cases, to evaluate the ferroptosis-mediated regulation of IR-driven ALI, we administered mice with ferrostatin-1 (Fer-1, 5 mg/kg; Sigma-Aldrich) via the caudal vein.

### Histology and scoring of lung injury

We fixed lung tissues in 10% formalin, embedded in paraffin, and sliced them into 5 µm slices, before staining with HE and observing with a digital camera (Optronics DEI-470; Goleta, CA) attached to a light microscope (Nikon-Ni-U; Japan). ALI was analyzed using a five-point scale (0–4) involving multiple parameters, namely, alveolar and mesenchymal edema, intra-alveolar inflammatory cell infiltrate, alveolar hemorrhage, and atelectasis. The grades were defined as follows: 0: normal, <15% of space is occupied by tissue (O-T) and >85% occupied by alveolar space (O-AS); 1: 15–25% of space is O-T and 75–85% is O-AS; 2: 25–50% is O-T and 50–75% is O-AS; 3: 50–75% is O-T and 25–50% is O-AS; and 4: 75–100% is O-T and 0–25% is O-AS.

### Transmission electron microscopy (TEM)

The lung tissues received a 2-h fixation in 0.05 M sodium cacodylate buffer with 2.5% glutaraldehyde at a pH of 7.2 at 25 °C. Next, they were incubated in 0.1 M sodium cacodylate buffer with 2% OsO4 for 2 h and, then, in 1% aqueous uranyl acetate for 18 h. Following sequential ethanol-induced dehydration, the specimens were embedded in Epon 812 and cut into ultrathin sections using copper grids and stained with uranyl acetate and lead citrate before visualization under a Tecnai G2 spirit BioTwin transmission electron microscope (FEI Company, Hillsboro, Oregon).

### Cell culture, OGD/R model procedures

MLE12 cells were acquired from the cell bank of the Chinese Academy of Sciences (Shanghai, China) and grown in an incubator at 37 °C and 5% CO_2_ in Dulbecco’s modified Eagle’s medium (11965, Gibco, USA) with 10% fetal bovine serum (0500, Gibco, USA), penicillin (100 IU/mL), and streptomycin sulfate (100 μg/mL).To create a model of oxygen–glucose deprivation and reoxygenation (OGD/R), the cells were cultured in glucose-free DMEM (TBI; China). The cells were washed in PBS supplemented with 0.5 mM CaCl_2_ and 1 mM MgCl_2_ and placed in an anaerobic chamber (5% CO_2_, 95% N_2_; Memmert; Schwabach, Germany) to induce OGD. After 8 h, the medium was replaced with normal culture medium and the plates were incubated in a normoxic chamber (37 °C, 5% CO_2_) for 12 h of reoxygenation.

### Cell viability assay

Cell viability was assessed with the Cell Counting Kit-8 (CCK-8, CK04, Dojindo, Tokyo, Japan), as per operational guidelines. In short, 5000 cells/well were seeded in 96-well plates and incubated for 24 h. Next, they were exposed to indicated treatments for a specified amount of time, at the end of which 10 μL CCK8 reagent was introduced to the cells to stain viable cells, followed by incubation at 37 °C for 3 h. Optical density (OD) was then measured at 450 nm with a microplate reader. Viable cells are expressed as percentage and compared against control cells. All experiments were conducted in triplicates.

### Western blotting

Total protein was isolated by lysing cells with RIPA lysis buffer supplemented with protease inhibitor cocktail. Protein quantification was done with BCA protein assay kit (PC0020, Solarbio, China). Equal quantities of protein samples were then mixed with loading buffer, heated for 10 min at 100 °C, separated on 10% SDS-PAGE gels, and subsequently transferred to PVDF membranes, which were then blocked with 5% nonfat milk for 1 h, exposed overnight (O/N) to specified antibodies at 4 °C, followed by three 15-min TBST rinses, and further exposure to anti-mouse or anti-rabbit horseradish peroxidase (HRP)-conjugated secondary antibody (2° Ab, 1:5000) at room temperature (RT) for 1 h, before visualization of the protein bands with ECL reagent and Amersham Imager 600 (General Electric Company, USA). Finally, the protein bands were quantified with Image J gel analysis software. All experiments were repeated three times. Among the primary antibodies (1° Abs) used were: Rabbit monoclonal anti-TERT (ab191523, Abcam, 1:1000); anti-Nrf2 (ab137550, Abcam, 1:1000); anti-SLC7A11 (ab37185, Abcam, 1:1000); anti-Ferritin (ab75973, Abcam, 1:1000); anti-Ferroportin (ab239583, Abcam, 1:1000); anti-GPX4 (ab125066, Abcam, 1:1000); and anti-β-Actin (4970S, Cell Signaling Tech, 1:1000).

### Quantitative real-time PCR

The total RNA was isolated using TRIzol reagent following the manufacturer’s protocol (Life Technologies) and subjected to cDNA synthesis using a Prime Script RT-PCR kit (TAKARA Korea, Seoul, Korea). A real-time RT-PCR analysis was performed using SYBR mix with a CFX384 real-time system (BioRad, Hercules, CA, USA). The following real-time PCR primers were used in the present study: Nrf2 upstream: 5′-TAGAGTCAGCAACGTGGAAG-3′ and downstream: 5′-TATCGAGGCTGTGTCGACTG-3′; TERT upstream: 5′-TTTCCTTCCACCAGGTGTCATC-3′, and downstream: 5′-AGCCAAAAGCCAGCACATTC-3′. SLC7A11 upstream: 5′-GCTGACACTCGTGCTATT- 3′ and downstream: 5′-ATTCTGGAGGTCTTTGGT-3′.

### Transient transfection

For overexpression and inhibition analysis, we acquired the M_Tert-shRNA3 (PGMLV-SC5); PGMLV-CMV-M_Tert-3×Flag-PGK-Puro; M_Nrf2(NFE2L2)-shRNA2(SB3); CMV-MCS-M_Nrf2-3×Flag-PGK-Puro; M_SLC7A11-shRNA3(SB3); and CMV-M_SLC7A11-3×Flag-PGK-Puro lentiviral vectors from Genomeditech (Shanghai, China). The purchased vectors were incorporated into MLE12, whereas the corresponding scramble lentivirus and empty vectors served as negative controls. To generate stable gene-overexpression and gene-silenced cell lines, we seeded 2.5 × 10^5^ cells/well into six-well plates and cultured until 40–50% confluency before incorporating corresponding vectors using lentiviruses, as per operational guidelines. Next, stably incorporated cells were selected with 2 μg/mL puromycin (1299MG025, BioFroxx, Germany). The overexpression and silencing efficiencies were validated with western blotting and qRT-PCR 72 h after vector incorporation; the efficiency was obvious and was shown in the subsequent figures

### MDA assay

MDA generation in tissues and cells were monitored following the MDA Assay Kit (D799762, Sangon Biotech, Shanghai, China) instructions.

### GSH assays

GSH was evaluated with a total GSH assay kit (BC1175, Solarbio, China), as per kit guidelines. In brief, lung tissues (100 mg) or cells (5 × 10^6^) were rinsed in chilled PBS before homogenization on ice in solution 1, followed by centrifugation at 8000 × *g* for 10 min at 4 °C to retrieve the supernatant for the GSH assay. Twenty microliters of samples were pipetted into a 96-well plate, followed by incubation at RT for 2 min with 140 µL solution 2 and solution 3, along with standards. Absorbance was read at 412 nm with a microplate reader. Finally, GSH levels were calculated according to the standard curve and normalized to protein levels, quantified by the Bradford protein assay. All experiments were done in triplicates.

### Fe assays

Intracellular total, ferric, and ferrous Fe levels was determined with the Fe assay kit (ab83366, Abcam), as per the operational guidelines. In short, lung tissues or cells were rinsed in chilled PBS before homogenization on ice in Fe assay buffer with a Dounce homogenizer, followed by centrifugation at 16,000 × *g* for 10 min, before collection of supernatant for Fe assay. Twenty-five microliter samples were then diluted to 100 µL in a 96-well plate with assay buffer, before incubation with 5 µL Fe reducer (for total Fe) or assay buffer (for ferrous Fe) along with standards at 37 °C for 30 min. Subsequently, 100 µL Fe probe was introduced to each well, mixed well, and maintained at 37 °C without light for 1 h. Absorbance was read at 593 nm with a microplate reader. Finally, Fe levels were calculated according to the standard curve and normalized to protein levels, quantified by the Bradford protein assay. All experiments were done in triplicates.

### Evaluation of Fe distribution in cells by STXM

4 × 10^5^ cells were plated in six-well plates with 100 nm Si_3_N_4_ membranes and incubated for 48 h. Next, the cells were fixed in 4% paraformaldehyde (PFA) for 15 min, and dehydrated in a series of ethanol dilution (30, 50, 70, 80, 90, and 95%), before detecting Fe with a soft-X-ray STXM on beamline 08U1A in Shanghai Synchrotron Radiation Facility [26]. The L_3_ absorption edge of Fe is chosen to analyze the distribution and content of Fe in the cell to be tested. The same cell was imaged twice with different incident photon energies above and below the absorption edge of Fe, respectively. Consequently, the Fe distribution map could be established by comparing the different absorption with incident beam.

### Statistical analysis

Data are presented as mean ± SEM. Intergroup comparisons were done with unpaired two-sided Student’s *t*-test and multi-group comparisons were done with one-way ANOVA test, followed by the Bonferroni post hoc test. A two-way ANOVA was employed for the evaluation of the outcomes between two independent variables. *P* < 0.05 was adjusted as the significance threshold. Lastly, SPSS statistical software 20.0 for Windows was employed for all analyses.

## Data Availability

The datasets generated and/or analyzed during the current study are available from the corresponding author on reasonable request.
